# Effect of Roadside Vegetation Cutting on Moose Browsing

**DOI:** 10.1371/journal.pone.0133155

**Published:** 2015-08-05

**Authors:** Amy L. Tanner, Shawn J. Leroux

**Affiliations:** Department of Biology, Memorial University of Newfoundland, St. John’s, NL, Canada; Université de Sherbrooke, CANADA

## Abstract

Moose (*Alces americanus* ) vehicle collisions (MVCs) are an issue throughout the distribution of moose. Many mitigation strategies have been tested and implemented to reduce the number of MVCs, but there have been few empirical analyses of the effectiveness of roadside vegetation cutting. The goal of this study was to determine if roadside vegetation cutting attracted moose into roadside areas to browse on the vegetation regrowth. We hypothesized that moose would be attracted to roadside areas with cut vegetation. Consequently, we predicted that there would be higher levels of browsing in cut areas compared to uncut areas. To determine if moose were browsing more in cut or uncut areas, we measured the number of plants browsed by moose in paired treatment (cut on or after 2008) and control (not cut since at least 2008) sites, along with a suite of potential environmental covariates. Using a model selection approach, we fit generalized linear mixed-effects models to determine the most parsimonious set of environmental variables to explain variation in the proportion of moose browse among sites. In contrast to our hypothesis, our results show that the proportion of moose browse in the uncut control areas was significantly higher than in the cut treatment areas. The results of this study suggest that recently cut roadside areas (7 years or less based on our work) may create a less attractive foraging habitat for moose. The majority of the variance in the proportion of moose browse among sites was explained by treatment type and nested plot number within site identification (34.16%), with additional variance explained by traffic region (5.00%) and moose density (4.35%). Based on our study, we recommend that vegetation cutting be continued in roadside areas in Newfoundland as recently cut areas may be less attractive browsing sites for moose.

## Introduction

Wildlife-vehicle collisions are a significant problem in many areas of the world, including the United States, Canada, and Europe [1–3]. As more roads and infrastructure are constructed, we decrease the natural connectivity of ecosystems, leading to wildlife-vehicle encounters. Large ungulates are one of the most problematic species groups involved in wildlife-vehicle collisions. The population size of ungulates in many areas is quite high, with over 1.1 million moose [4] and 28.5 million white-tailed deer [5] occurring in North America alone. These large population sizes paired with an expanding road network increase the likelihood of ungulates being near roads and therefore being involved in wildlife-vehicle collisions. The primary issues associated with a high ungulate population and collisions with vehicles are injuries to humans and damage to property resulting from the large physical size of ungulates. High levels of wildlife-vehicle collisions, particularly ungulate-vehicle collisions, cause a concern for the public’s safety, prompting the implementation of mitigation strategies to reduce the number of collisions [6].

There are many different mitigation strategies that have been designed and implemented in an attempt to reduce the number of wildlife-vehicle collisions around the world (see review in Huijser, Duffield [6]). Common mitigation strategies include physical barriers, deterrents, and public awareness programs. Physical barriers, such as fences along the edge of the highway, seek to preclude access to the roadway [7, 8]. Deterrents, such as warning reflectors, seek to make crossing the road undesirable for wildlife [9–11]. Public awareness programs, such as wildlife crossing signs [12, 13] and cutting roadside vegetation [14], inform drivers about the increased risk for wildlife in certain area. Each mitigation strategy has pros and cons and different strategies are more likely to be successful under different conditions. Wildlife fencing, for example, has reduced wildlife-vehicle collisions in many areas because it prevents wildlife from trying to cross the road [8, 15]. However, in addition to wildlife fencing being an expensive mitigation strategy, it has further negative consequences such as trapping animals within the fenced area, and acting as a barrier to animal movement, consequently reducing gene flow across landscapes [16, 17]. It is therefore important to conduct both research and monitoring on different mitigation strategies to see which are the most effective in specific environments. While many mitigation strategies have been widely studied, one common mitigation strategy, roadside vegetation cutting, which involves clearing or cutting vegetation along roadsides to improve driver visibility of animals near roads [18–20] (also referred to as roadside brush cutting), has had minimal empirical analysis. This project was designed to be a first step to investigate if cutting of roadside vegetation attracted moose into roadside areas to feed in Newfoundland, Canada.

Roadside vegetation is being cut in Newfoundland and in many other areas across Canada, allowing drivers an opportunity to see wildlife in the roadside areas and adjust their driving to avoid collisions [21]. However, roadside vegetation cutting could have unintended consequences because while it increases driver visibility, cutting may actually be attracting moose to the roadside areas to feed on the new vegetation growth [14]. Continual cutting of roadside vegetation prevents forest succession from occurring [14], which leaves the ecosystem in an early successional state and provides optimal moose foraging habitat [22].

The goal of this study is to determine if roadside vegetation cutting attracts moose into roadside areas to browse on the vegetation regrowth. Hughes and Fahey [23] indicate that ungulates prefer to feed on plant regrowth for at least the first 3 years after cutting, or new plant growth, due to its high nutritional content. If roadside vegetation regrew with suitable moose forage, then we expect moose to spend more time near roads, and consequently pose a higher risk for moose-vehicle collisions (MVCs). Specifically, we hypothesize that moose are attracted to roadside areas with cut vegetation rather than to areas where no vegetation cutting has occurred in at least the past 7 years (based on access to roadside vegetation cutting data). Consequently, we predict that there will be higher levels of browsing in cut areas compared to uncut areas. We test this hypothesis by comparing the amount of moose browse occurring in areas where roadside vegetation has been recently cut to areas where it has not been recently cut.

## Study Area

**Fig 1:**
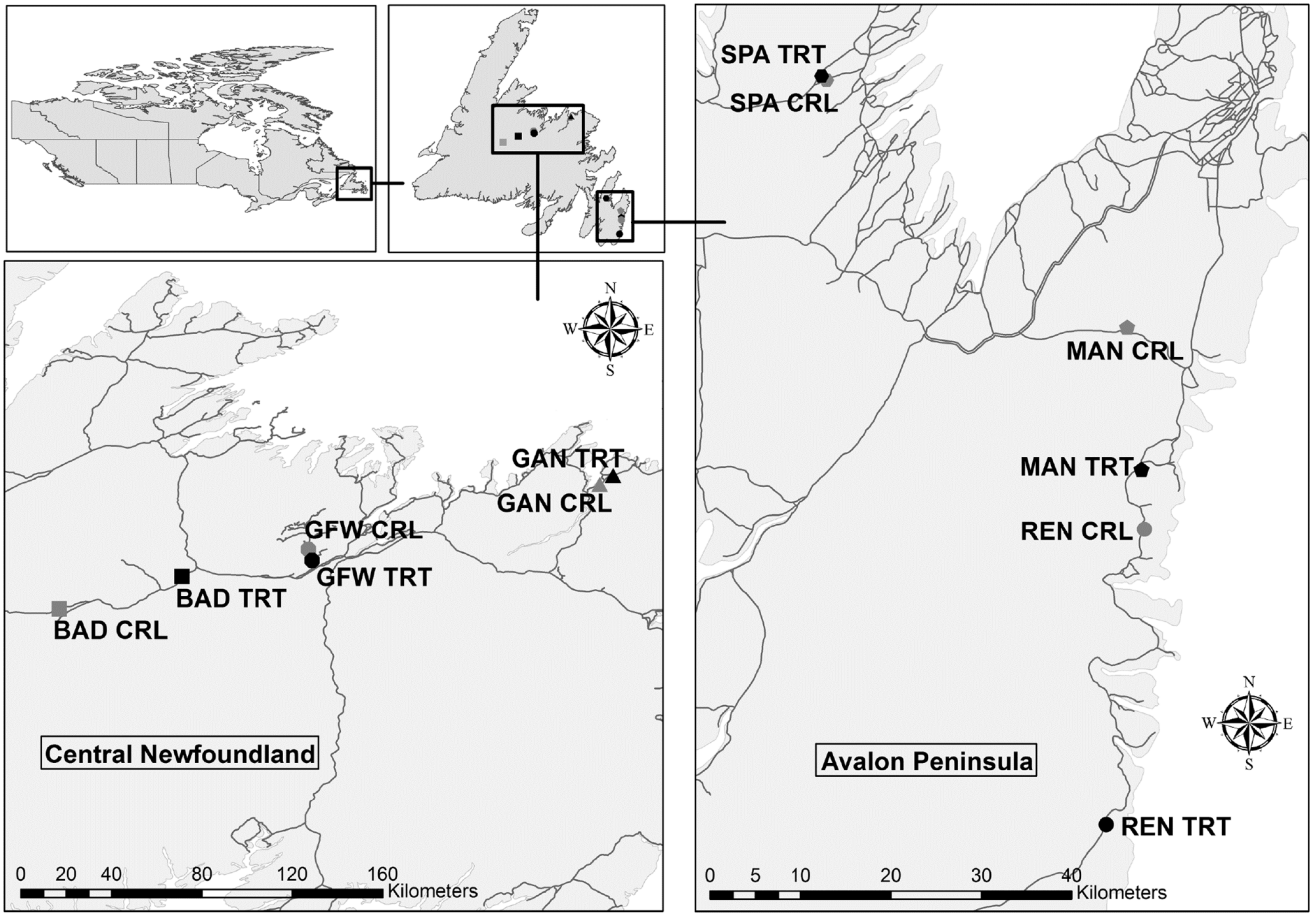
Map of study sites. The locations of the paired treatment (TRT, black points) and control (CRL, gray points) locations used in our study in Newfoundland, Canada. The linear gray features are roads. Vegetation and evidence of moose browse at the sites was sampled from June 17^th^–July 23^rd^ 2014. Locations; BAD: Badger, GFW: Grand Falls-Windsor, GAN: Gander Bay, MAN: La Manche Provincial Park, REN: Renews-Cappahayden, and SPA: Spaniards Bay.

Newfoundland is an island in the North Atlantic Ocean that falls within the boreal forest region. The study was conducted from June 17^th^ to July 23^rd^ 2014 along roadsides in two ecoregions in Newfoundland, Canada: maritime barrens and central Newfoundland forest. The sites in the maritime barrens region, on the Avalon Peninsula, were La Manche Provincial Park (MAN), Renews-Cappahayden (REN), and Spaniard’s Bay (SPA) (Fig 1). The region is dominated by black spruce (*Picea mariana*), balsam fir (*Abies balsamea*), tamarack (*Larix laricina*) and many shrub and lichen species, with a mean annual precipitation of 1,400 mm and temperature of 5.5°C [24]. The sites in the central Newfoundland forest region were Badger (BAD), Grand Falls-Windsor (GFW), and Gander Bay (GAN) (Fig 1). The region is dominated by black spruce, balsam fir, paper birch (*Betula papyrifera*), trembling aspen (*Populus tremuloides*), and sheep laurel (*Kalmia angustifolia*), with a mean annual precipitation of 1,150 mm and temperature of 4.5°C [25].

## Methods

### Site Selection

All vegetation adjacent to the road in Newfoundland is cut back approximately 20 m along main roads, such as the Trans-Canada Highway (TCH). We paired treatment sites with nearby control sites that had similar biophysical traits (i.e. no herbicide use after 2009, elevation, road speeds, vegetation cut widths, traffic volumes, and moose densities), but differed in the age of cut vegetation (Table 1, S1 and S2 Tables). We obtained data from the Department of Transportation and Works for roadside vegetation cutting projects issued by the Government of Newfoundland from 2008 to 2013 and herbicide application projects from 2010 to 2013. Herbicide application and vegetation cutting data were unavailable for the paired control sites (hereafter controls) prior to 2010 and 2008 respectively. We selected secondary roads for our sampling due to the high traffic volume and associated risk of sampling beside a busy highway. The side of the road to be sampled was randomly selected except if there was additional infrastructure making one side unsuitable for our study (e.g. power lines). The field study did not involve the sampling of endangered or protected species and was conducted on crown land, which does not require permits or approvals in Newfoundland or Canada.

**Table 1:** Descriptive statistics of explanatory variables.

		Treatment type					
Variable	Description	Control		2008–2010 Cut		2011–2013 Cut	
		mean	SD	mean	SD	Mean	SD
Water bodies	Presence or absence of water bodies (0 = no water, 1 = yes water)	0.17	±0.38	0.67	±0.48	0.67	±0.48
Width	Width of site (m)	14.17	±1.56	15.07	±1.01	13.27	±1.50
Road speed	Road speed limit (km/h)	71.67	±12.25	70.00	±14.41	80.00	±0.00
Gradient road	Gradient up to roadside (cm)	0.43	±0.15	0.49	±0.05	0.62	±0.06
Gradient tree	Gradient up to tree-side (cm)	0.31	±0.25	0.05	±0.07	0.15	±0.22
Traffic region	Traffic region (0 = Avalon, 1 = Central Newfoundland)	0.50	±0.50	0.33	±0.48	0.67	±0.48
Moose density	Moose density (# moose/ km^2^)	2.16	±1.09	2.21	±1.09	2.10	±1.12
Elevation	Elevation (m)	106.50	±60.19	86.00	±32.68	70.33	±27.22
Proportion of preferred plants	Plant preference index (# of preferred plants per plot/ total # of plants per plot)	0.51	±0.32	0.34	±0.25	0.31	±0.27

Descriptive statistics for the explanatory variables included in the correlation analysis of the proportion of moose browsed plants in roadside areas. Data were collected from June 17^th^–July 23^rd^ 2014 in roadside sampling locations in Newfoundland, Canada.

### Data Collection

**Fig 2:**
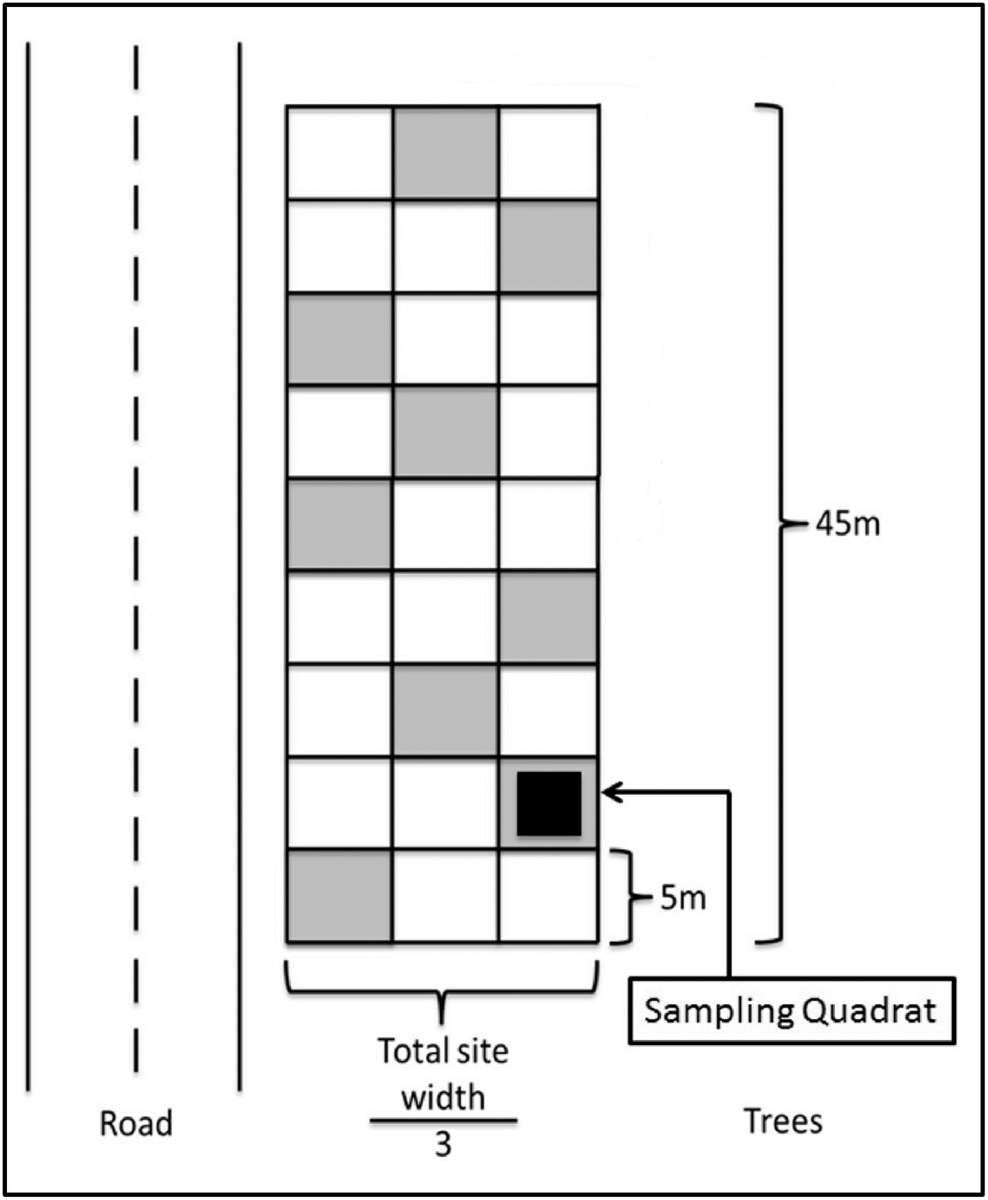
Overview of field sampling design. Schematic of sampling grid used to select sampling plots within sites for quantifying the proportion of plants browsed by moose in Newfoundland, Canada. We used stratified random sampling and the gray boxes represent one potential set of plots sampled at a site. The total width of the site was divided by 3 to ensure that there were 3 rows of sampling. The black box represents the 9-m^2^ quadrat that was sampled within each of the gray plots.

We used a stratified random sampling grid to measure the number of plants browsed by moose per plot in roadside areas with cut vegetation and in nearby control areas [26]. A 45 m long grid was laid out parallel to the roadway and subset into 9 5-m sections. The width of the sampling grid was determined by the width of the roadside vegetation cut area at each site. We divided the width into 3 equal sections, giving us 27 potential plots to sample per grid. We randomly selected 9 plots, one in each 5-m section, making sure to avoid having spatially adjacent plots (Fig 2). We sampled vegetation in a 9-m^2^ quadrat placed in the center of each of the 9 plots per site.

To determine the amount of moose browse, we measured a series of plant traits in each 9-m^2^ quadrat. An overall percent ground cover was visually determined for each site. Woody plants within the 9-m^2^ quadrats were identified to species. Evidence of moose browse is readily detectable on woody plants [27]; allowing us to record whether or not the woody plant had been browsed by moose (i.e., we measured moose browse as a binary response—browsed or not browsed). We also recorded the height of each plant in our plots. We collected data on road speed limit, presence of water bodies within the site, and the gradient of the site.

The response variable in this analysis was the proportion of moose browsed plants per plot measured as (the number of browsed plants/ the total number of browsable plants per plot) on an annual scale. We considered plants that were browsed at least once by moose in the entire study as browsable. We measured a series of discrete and continuous variables that may influence moose browse along roadsides. The discrete explanatory variables were: treatment type, presence or absence of water bodies, and traffic region (S3 Table). Treatment type was a categorical variable with three levels; control (not cut since at least 2008), treatment 1 (cut between 2008–2010), and treatment 2 (cut between 2011–2013). Presence or absence of water bodies was determined visually within each site during sampling, and we split the study sites into two traffic regions based on the difference in average daily traffic, i.e., the Avalon and central Newfoundland. The Avalon Peninsula is located on the south-eastern edge of Newfoundland and contains the capital of St. John’s (Fig 1). Traffic counters deployed at the sites from June 11^th^ to July 1^st^ 2014 indicated that sites on the Avalon Peninsula experienced much higher traffic volumes (mean ±SD number of vehicles per day: 1,889 ±275) than sites in central Newfoundland (mean ±SD number of vehicles per day: 670 ±242).

We included 6 continuous explanatory variables for variation in the proportion of browsed plants along roadsides: the width of the site, gradient up to the roadside, gradient up to the tree-side, road speed limit, moose density, and plant preference index (S3 Table). Width of the site was measured in the center of the site, from where continuous vegetation started closest to the road up to the edge of the tree line. The gradient up to the roadside was measured as the mean slope of the site from the bottom of the site towards the road from points taken on either end and in the center of the site. The gradient up to the tree-side was measured in a similar manner as the gradient up to the roadside except it was measured from the bottom of the site and toward the trees. Road speed limit was determined using the posted speed limit signs on each road. Moose density was calculated for each moose management area using a stratified-random block aerial survey design, conducted by the Department of Environment and Conservation Wildlife Division [28]. Each moose management area is stratified and all moose and tracks recorded, then blocks are assigned to low, medium, or high moose density categories. A sightability correction factor is then applied to each category based on land cover and topography of the survey area. Different moose management areas are surveyed every year with an effort being made to have at least one moose management area in each of the island ecoregions surveyed per year.

### Statistical Analysis

#### Moose plant preference

We attempted to control for differences in plant “quality” or preference across sites by selecting control areas close to the treatment areas. While many plants may be only occasionally browsed, preferred species are consumed in a larger proportion than their availability in the environment [29]. Most other studies present a list of plant species that they deem to be preferred or high quality without any justification of the distinction between preferred and non-preferred species (e.g., [30–32]). We used our browse data to define what is considered a preferred resource for Newfoundland moose. Plants that were browsed at least once by moose in the entire study were used in the analysis to determine the proportion of browsable plants browsed, with the proportion calculated as (the number of browsed plants of species i/ the total number of plants of species i). Then, to identify a potential preference or quality threshold in plant species used by moose in Newfoundland, we applied segmented regression (segmented package in R v.3.0.1 [33]) to the frequency of the plants that were browsed at least once by moose in our study. The segmented regression identified a threshold in browse frequency whereby plants above the threshold are browsed more frequently than plants below the threshold. We considered plants above this threshold as preferred moose browse. The quality of each site, determined via the plant preference index, was then calculated as (the number of preferred plants per plot/ the total number of plants per plot) (S1 Fig).

#### Model selection for proportion of browsed plants by moose

Using a model selection approach, we built generalized linear mixed-effects models with a hierarchical structure, containing a logit canonical link and a binomial error structure. We included plots nested within sites as random variables in all of our models to account for the hierarchical structure of our sampling and our paired treatment-control site design. We also included sites as a grouping variable to account for some of the variation in plant presence among sites. Proportion of browsed plants was the dependent variable and we had a suite of 3 discrete and 6 continuous explanatory variables (S3 Table). As explanatory variables that are highly correlated with each other should not be included in the same model, we conducted both Pearson’s and Spearman’s correlation analysis to determine which explanatory variables to include as fixed effects in our models (S4 Table). Since the main goal of the study was to investigate whether vegetation cutting altered the proportion of plants browsed by moose, we decided a priori to include treatment type as an explanatory variable in all of the potential models. The only variables not highly correlated with treatment type, and therefore the only other variables included in our models, were traffic region, width of site, and moose density. Treatment type was significantly correlated with site quality, with control sites having higher browse quality than cut sites (*rho* = −0.30, S = 272145.8, *P* = 0.002) (S2 Fig). Because these variables are correlated, we are unable to determine the relative importance of treatment type versus site quality in explaining variation in moose browse along roadsides. However, we fit generalized linear mixed-effects models of proportion of moose browse and treatment type, and proportion of moose browse and site quality (based on the plant preference index) to determine which variable was the most parsimonious predictor of moose browse. Additionally, we included a weighted vector of the number of plants per plot to account for the differences in the number of plants among plots. We used the glmer function within the lme4 package [34] in R v.3.0.1 for all of our analysis. The R code and associated data are available on figshare [35].

We used Akaike Information Criterion corrected for small sample size (AIC_c_) to determine the most parsimonious model out of all of the competing models. We considered any model with a ΔAIC_c_<2 as a parsimonious model [36]. We calculated the amount of variance explained by each variable by calculating the improvement in the marginal R^2^ value when these additional variables were added to the basic model. Width was a potential variable that was included in the original model set, but was a pretending variable (sensu Anderson [37]) and therefore the two models containing width were removed. Pretending variables do not explain additional variation in the model but their inclusion in the candidate set of models can erroneously increase model selection uncertainty [37].

## Results

### Moose Plant Preference

**Fig 3:**
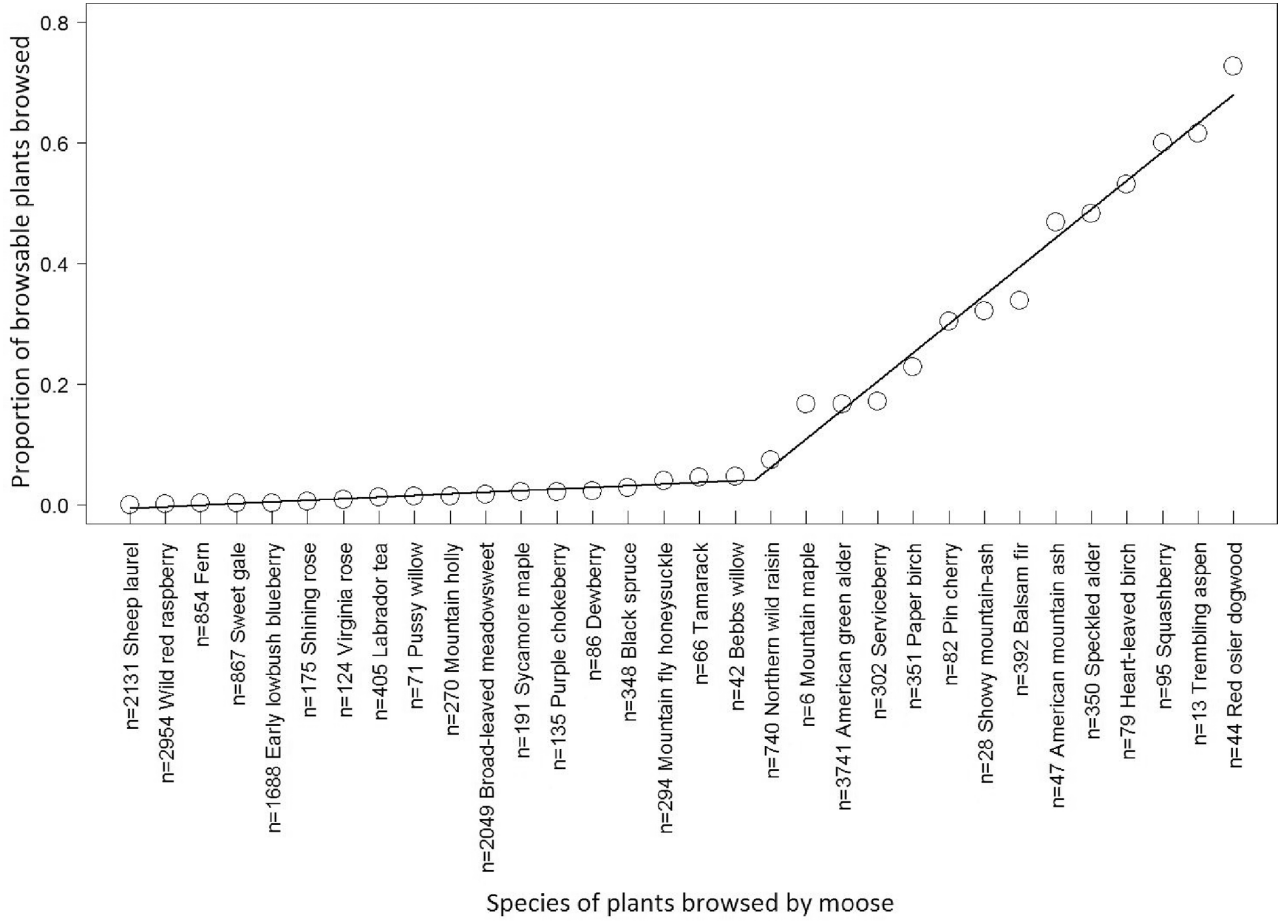
Segmented regression analysis of moose plant preference. Segmented regression analysis used to determine preferred plant species for moose within the entire study. The proportion of browsable plants browsed was determined using plants that were browsed at least once by moose in the entire study and then calculated as (the number of browsed plants of species i/ the total number of plants of species i). The threshold occurs after Bebb’s willow (*Salix bebbiana*), indicating that all plants from northern wild raisin (*Viburnum nudum*) through red osier dogwood (*Cornus stolonifera*) are considered preferred forage species for moose in our study area. The n value before each plant name indicates the total number of individuals in all plots. Equation and R^2^ for each line segment (line 1: *y = 0*.*0027x−0*.*0082*, R^2^ = 0.91; line 2: *y = 0*.*0475x−0*.*8398*, R^2^ = 0.98).

Of the 32 plant species that showed at least one occurrence of moose browse in the study, 18 species show very low frequency of moose browse (mean = 1.74%, range = 0.05% to 4.76% of individual plants were browsed) and 14 species showed relatively high frequency of moose browse (mean = 37.14%, range = 7.43% to 72.73% of individual plants were browsed) (Fig 3). Our segmented regression of browse frequency identified a single threshold (i.e. a shift in frequency of moose browse) in the proportion of moose browse at 4.76% browse (Fig 3). Consequently, we considered plants with more than 4.76% browse to be preferred plants for moose. In terms of abundance, wild red raspberry (*Rubus idaeus*) is abundant (n = 2954) but rarely selected when present, while trembling aspen is scarce (n = 13) but often selected when present (Fig 3).

### Model Selection for Proportion of Browsed Plants by Moose

The candidate set of models consisted of four models, with three of the models having ΔAIC_c_ values of <2 and ωAIC_c_ between 0.26 and 0.38 (Table 2), indicating that they were parsimonious models for explaining variation in the proportion of moose browse in roadside areas. These top models included the fixed effects variables: treatment type, traffic region, and moose density, and the nested random effects: site identification and plot number (Table 2). These models explained between 34% and 39% of the variation in the proportion of moose browse in roadside areas. Our basic model consisted of treatment type and plot number nested within site identification, which explained the majority of the variance (34.16%, Table 2) in the proportion of moose browse among sites. Traffic region (5.00%, Table 2) and moose density (4.35%, Table 2) explained a much lower amount of variance in the proportion of moose browse among sites. A comparison of models with either treatment type or plant preference showed that treatment type (ωAIC_c_ = 1.00) was a more parsimonious explanatory variable for variation in moose browse along roadsides than preferred species (ωAIC_c_ = 0.00) (S5 Table). Treatment type, traffic region, and moose density were all negatively correlated with the proportion of moose browse in roadside areas (Table 3).

**Table 2:** Results of model selection examining the effect of roadside vegetation cutting and environmental variables on moose browsing.

Model^a^	Description	k^b^	LL^b^	Marginal *R* ^2^ ^b^	Conditional *R* ^2^ ^b^	ΔAIC_c_ ^b^	ωAIC_c_ ^b^
1	treatment type + moose density	6	−336.93	0.39	0.43	0.00	0.38
2	treatment type + traffic region	6	−337.01	0.39	0.44	0.16	0.35
3	treatment type	5	−338.32	0.34	0.42	0.78	0.26
4	null model	3	−371.17	0.00	0.15	62.48	0.00

Four generalized linear mixed-effects models included in model selection to determine which environmental explanatory variables influenced the proportion of moose browsed plants along roadsides. The variables plot number nested within site id were included as random effects in all models.

^a^ Models are ranked with Akaike Information Criterion, corrected for small sample size (AIC_c_)

^b^ Key: k, number of parameters; LL, log-likelihood; Marginal *R* ^2^, Nakagawa and Schielzeth’s Marginal *R* ^2^ where the fixed factors alone explain the proportion of variance; Conditional *R* ^2^, Nakagawa and Schielzeth’s Conditional *R* ^2^ where both the fixed and random factors explain the proportion of variance; ΔAIC_c_, the difference in the AIC_c_; ωAIC_c_, model weights.

**Table 3:** Parameter estimates (95% CI) of generalized linear mixed-effects models of roadside moose browse.

Fixed effect	Parameter estimate	95% CI
Model 1: proportion browsed plants ~ treatment type + moose density		
Intercept (control)	-0.84	
Treatment type (2008–2010)	-1.58	-2.54, -0.61
Treatment type (2011–2013)	-4.77	-6.03, -3.51
Moose density	-0.59	-1.20, 0.01
Model 2: proportion browsed plants ~ treatment type + traffic region		
Intercept (control)	-1.48	
Treatment type (2008–2010)	-1.68	-2.63, -0.74
Treatment type (2011–2013)	-4.63	-5.86, -3.41
Traffic region (Yes: on Avalon)	-1.25	-2.62, 0.12
Model 3: proportion browsed plants ~ treatment type		
Intercept (control)	-2.10	
Treatment type (2008–2010)	-1.67	-2.62, -0.71
Treatment type (2011–2013)	-4.67	-5.91, -3.43
Model 4: proportion browsed plants ~ 1		
Intercept (control)	-3.57	

Results of the 4 generalized linear mixed-effects models used to determine which variables influenced the proportion of moose browse within the treatment and control sites in Newfoundland, Canada.

**Fig 4:**
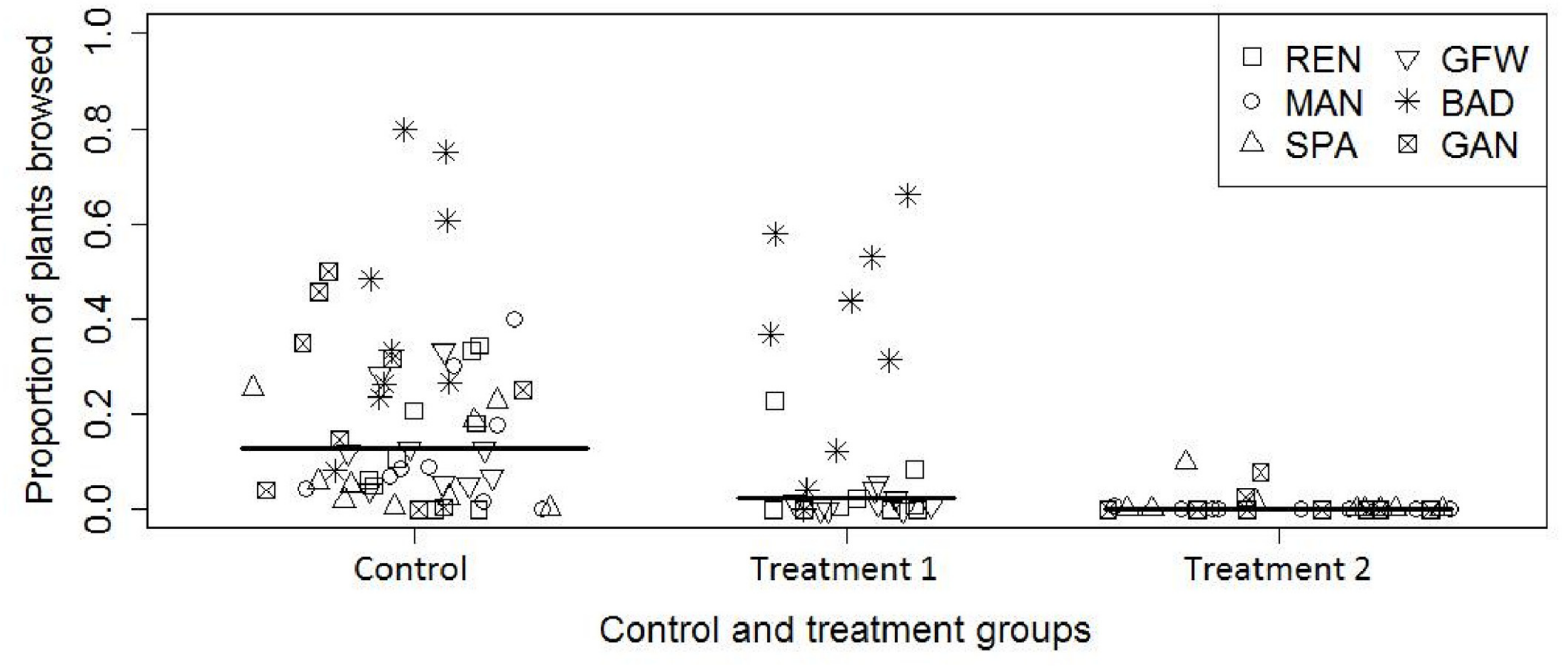
Proportion of plants browsed in each treatment and control site. The proportion of plants browsed by moose in all of the control sites (Control) vs all of the treatment sites cut from 2008–2010 (Treatment 1) vs all of the treatment sites cut from 2011–2013 (Treatment 2). Each point represents data from a single plot. The solid black lines represent the median proportion of browsed plants in each of the control (median = 0.13), treatment 1 (median = 0.02), and treatment 2 (median = 0.00) groups. For the locations; BAD: Badger, GFW: Grand Falls-Windsor, GAN: Gander Bay, MAN: La Manche Provincial Park, REN: Renews-Cappahayden, and SPA: Spaniards Bay.

The proportion of moose browse in the control areas was 5.67 times higher than the proportion of moose browse in the 2008–2010 cut treatment areas and it was considerably higher than proportion of browse in the 2011–2013 cut treatment areas (Fig 4). The proportion of moose browse in the 2008–2010 cut treatment areas was also higher than the proportion of browse in the 2011–2013 cut treatment areas (Fig 4). The percent of vegetation within 3 different height categories (<30 cm, 30–200 cm, and >200 cm) was found to be comparable between both treatment and control sites (S6 Table).

## Discussion

Roadside cutting is often used as a method to improve visibility of moose on the sides of roadways, but it may have unintended consequences if cut areas act as an attractant for moose. Even though our study only contained six paired treatment-control sites, the effect size of roadside vegetation cutting on moose browsing was very large. Specifically, the proportion of plants browsed by moose in the control sites was on average 1.5 to 23.2 times higher than in the two treatment areas—this despite the sites having similar vegetation communities. These results, which are in contrast to our initial hypothesis, thereby suggest that recently cut areas may not act as attractants for moose browse.

Child, Barry [38] suggested that management of roadside vegetation creates favorable habitats for moose by maintaining early seral vegetation. Additionally, Rea [14] suggested that roadside vegetation cutting could unintentionally stimulate plant regrowth that is more nutritious, ultimately increasing the attractiveness of the area for moose foraging, and consequently increasing the likelihood of MVCs. However, contrary to our hypothesis based on previous work, we found evidence of more moose browse in control areas than in treatment areas. We also found that cutting treatment best explained the variance in the proportion of moose browse among sites (Table 2, Fig 4). These results indicate that roadside vegetation cutting does play a large role in the amount of moose browse occurring in roadside areas, which could have a direct effect on the number of MVCs occurring. We have not, however, investigated the effect of roadside vegetation cutting on the number of MVCs directly. Nevertheless, since our results are contrary to Rea [14], it follows that if roadside areas are less attractive to moose, then the likelihood of MVCs should decrease within cut areas especially since cutting is also performed to increase driver visibility [20, 39, 40]. Work by and Andreassen, Gundersen [41] and Jaren, Andersen [42] indicated that cutting vegetation along railways resulted in a 40% to 56% decrease in the number of moose-train collisions, respectively. Cutting of vegetation appears to be a successful mitigation strategy to reduce moose-train collisions in Norway and future studies can build on this and our work to determine if it is a successful mitigation strategy for other vehicle types in other locations.

The species and availability of plants in roadside areas will be a key determinant of moose browse potential (S3 Table). Many studies have independently determined preferred or high quality species for moose to browse on [30, 31, 43] but the species on the lists vary and there are no quick methods to differentiate preferred from non-preferred species. We were interested in identifying the types of species that moose forage on in roadside areas. Consequently, we developed a surrogate technique to rapidly determine plant species that are preferred by moose in place of more detailed and specific methods such as Dodds [43]. A clear threshold existed in our data where certain plant species could be considered frequently used resources for moose along secondary roads in Newfoundland (Fig 3). Dodds [43] determined the percent use of plants by moose in Newfoundland by examining the number of stems browsed by moose. Our method is far less time consuming as it only considers if the plant has been browsed by moose or not, rather than counting individual stems. The analysis provided consistent results for preferred or high quality browse species with respect to Dodds [43], as 12 of the 14 species determined to be preferred by our calculations were also included on Dodds’ list. We believe our threshold approach will be useful in other studies attempting to quantify resource quality from plot to landscape level, and will significantly reduce the sampling time required to identify similar species that are deemed preferred forage species by other more time consuming techniques.

Moose density also explained a small amount of the variance in moose browse along roadsides (Table 2). We included moose density because we hypothesized that it would play a role in the proportion of moose browse occurring. However, our model predicts that the proportion of roadside moose browse will decline with increasing moose densities (Table 3). This is contradictory to our expectation and it may be explained by the fact that the measure of moose density was too coarse (moose management areas within Newfoundland) to capture small scale variation in moose densities around secondary roads. An alternative explanation would be that areas with high moose density provide sufficient food and allow a large population of moose to thrive in the area without having to frequent roadside areas to browse. Additionally, traffic region (on Avalon or central Newfoundland) also explained a small portion of the variation in moose browse along roadside (Table 2). The model including traffic region predicts that the proportion of moose browse occurring in roadside areas will be lower in areas with more traffic (i.e. Avalon) (Table 3). Moose may avoid areas with higher traffic volume or reduce their crossing rates in regions with high traffic volume [32, 44, 45].

Our correlational study has low inferential strength, but it does provide a new line of evidence about roadside vegetation cutting and its effect on moose browsing. Our study could be improved by having true control areas that had never been cut and by having more treatment and control sites overall to increase the strength of our inference. Our data, however, does clearly indicate that roadside vegetation cutting of secondary roads in Newfoundland does not attract moose to roadside areas to browse on plant regrowth as previously suggested [14, 38]. Future studies could examine the direct links between roadside vegetation cutting and the probability of MVCs. This could be achieved by building on Joyce and Mahoney’s [46] large-scale spatial analysis of the determinants of MVCs. A revised analysis would make use of new georeferenced MVC data for the island of Newfoundland that were not available before 2012.

### Management Implications

We provide the first line of evidence that recently cut roadside areas may not be attractive browse areas for moose. Based on our study we recommend that vegetation cutting be continued in roadside areas to both increase driver visibility and to reduce the attractiveness of the area for moose to browse. In our case, sites cut between 1 to 7 years of our sampling had lower moose browse than control areas which suggests that a regime of frequent roadside cutting may help mitigate moose browse along roadsides. Additionally, our surrogate technique for determining preferred forage species will save considerable time in the field and can be applied to studies of ungulate browsing conducted outside of Newfoundland. The main issue of reduction of MVCs in Newfoundland will not, however, be achieved through the implementation of one mitigation strategy. We do not have data to speak beyond roadside clearing as a mitigation strategy but based on other work [6, 47], a comprehensive MVC reduction program should evaluate all possible strategies and the costs and benefits of each. In the end, a mitigation strategy that works in one area may not work in another due to multiple extenuating factors, including the physical landscape or general ecosystem structure. For example, underpasses were implemented in Alberta in combination with fences and resulted in substantial reductions in wildlife-vehicle collisions [8], but this strategy may not be feasible for many regions due to the bedrock being extremely close to the surface. All mitigation strategies adopted should be studied within an adaptive management framework [48], where the effectiveness of the strategy is carefully monitored and the strategies can be modified over time based on their “success”.

## Supporting Information

S1 Fig Comparison of the proportion of browsable plants that are browsed and preferred plants per plot. The proportion of moose browsed plants (dark gray) and the proportion of preferred plants (light gray) for each treatment (trt1 & trt2) and control (ctrl) site. Preferred plants are the 14 forage species that had significantly higher browse frequency as identified with our segmented regression analysis (see text for details). The locations; BAD: Badger, GFW: Grand Falls-Windsor, GAN: Gander Bay, MAN: La Manche Provincial Park, REN: Renews-Cappahayden, and SPA: Spaniards Bay.
(DOCX)

S2 Fig Correlation between the proportion of preferred plants per plot and the three treatment types. Displaying the correlation between the proportion of preferred plants per plot and the 3 treatment types. There were higher quality plants present in the control areas than in the treatment areas (*rho* = −0.30, S = 272145.8, *P* = 0.002). Since we were testing for the effect that roadside vegetation cutting had on the proportion of moose browse in roadside areas (and through further AIC_c_ analysis), treatment type was used as the main explanatory variable rather than preferred plants. For the control and treatment groups: control sites: not cut since at least 2008, treatment 1 sites: cut from 2008–2010, and treatment 2 sites: cut from 2011–2013. For the locations; BAD: Badger, GFW: Grand Falls-Windsor, GAN: Gander Bay, MAN: La Manche Provincial Park, REN: Renews-Cappahayden, and SPA: Spaniards Bay.
(DOCX)

S1 Table General description of field study sites. Site description including; GPS locations, road speed limit, width, presence of water bodies, moose density and the gradient for both the road and tree sides of the site for the cut treatment (TRT) and uncut control (CRL) sites collected from June 17 –July 23 2014 in Newfoundland, Canada. For the locations; BAD: Badger, GFW: Grand Falls-Windsor, GAN: Gander Bay, MAN: La Manche Provincial Park, REN: Renews-Cappahayden, and SPA: Spaniards Bay.
(DOCX)

S2 Table Descriptions of the field sites for the three treatment types. Pictures of one of our control, treatment 1, and treatment 2 sampling sites, showing the width of the cut (in the treatment areas) and the height of the vegetation. A black arrow indicates the location of a person (height: 5’6” or 1.68 m) as a reference for the height of the vegetation.
(DOCX)

S3 Table Hypothesis of the effect of each explanatory variable on the proportion of moose browse along roadsides. Potential hypotheses for the effect that each explanatory variable would have individually on the proportion of moose browse in the roadside areas in Newfoundland, Canada.
(DOCX)

S4 Table Pearson’s and Spearman’s correlation analyses of explanatory variables for moose browse along roadsides. Pearson’s and Spearman’s correlation analyses were performed to determine which explanatory variables to include as fixed effects in the models. Pearson’s correlation was performed when both variables were continuous and Spearman’s correlation was performed when either one or both variables were discrete. The tolerance for Type 1 error was set at α = 0.05, therefore variables were considered correlated if the p-value was <0.05.
(DOCX)

S5 Table Glmer’s for comparison of treatment type and proportion of preferred plants. Two generalized linear mixed-effects models used to determine if treatment type or preferred plants were a better predictor of the proportion of browsed plants. The variables plot number and nested within site id were included as random effects in both models.
(DOCX)

S6 Table Summary of plant height in the sampling sites. Descriptive statistics of plant height in the sampling sites (CTRL: control—not cut since at least 2008, TRT 1: treatment 1 –cut between 2008–2010, and TRT 2: treatment 2 –cut between 2011–2013) in Newfoundland. The chart provides an overview of the structure of the plant community, including the proportion of plants in 3 height categories, and 1 combined category. The distinction at 30 cm was made because moose rarely browse below this height [49].
(DOCX)
