# Supplementary material for: Effect of Roadside Vegetation Cutting on Moose Browsing
Source: PLoS One. 2015 Aug 5;10(8):e0133155. doi: 10.1371/journal.pone.0133155 (PMC4526696; doi:10.1371/journal.pone.0133155)
Supplement: S1 Fig — The proportion of moose browsed plants (dark gray) and the proportion of preferred plants (light gray) for each treatment (trt1 & trt2) and control (ctrl) site. Preferred plants are the 14 forage species that had significantly higher browse frequency as identified with our segmented regression analysis (see text for details). The locations; BAD: Badger, GFW: Grand Falls-Windsor, GAN: Gander Bay, MAN: La Manche Provincial Park, REN: Renews-Cappahayden, and SPA: Spaniards Bay. (DOCX) [file pone.0133155.s001.docx]

**S1 Figure. Comparison of the proportion of browsable plants that are browsed and preferred plants per plot.**
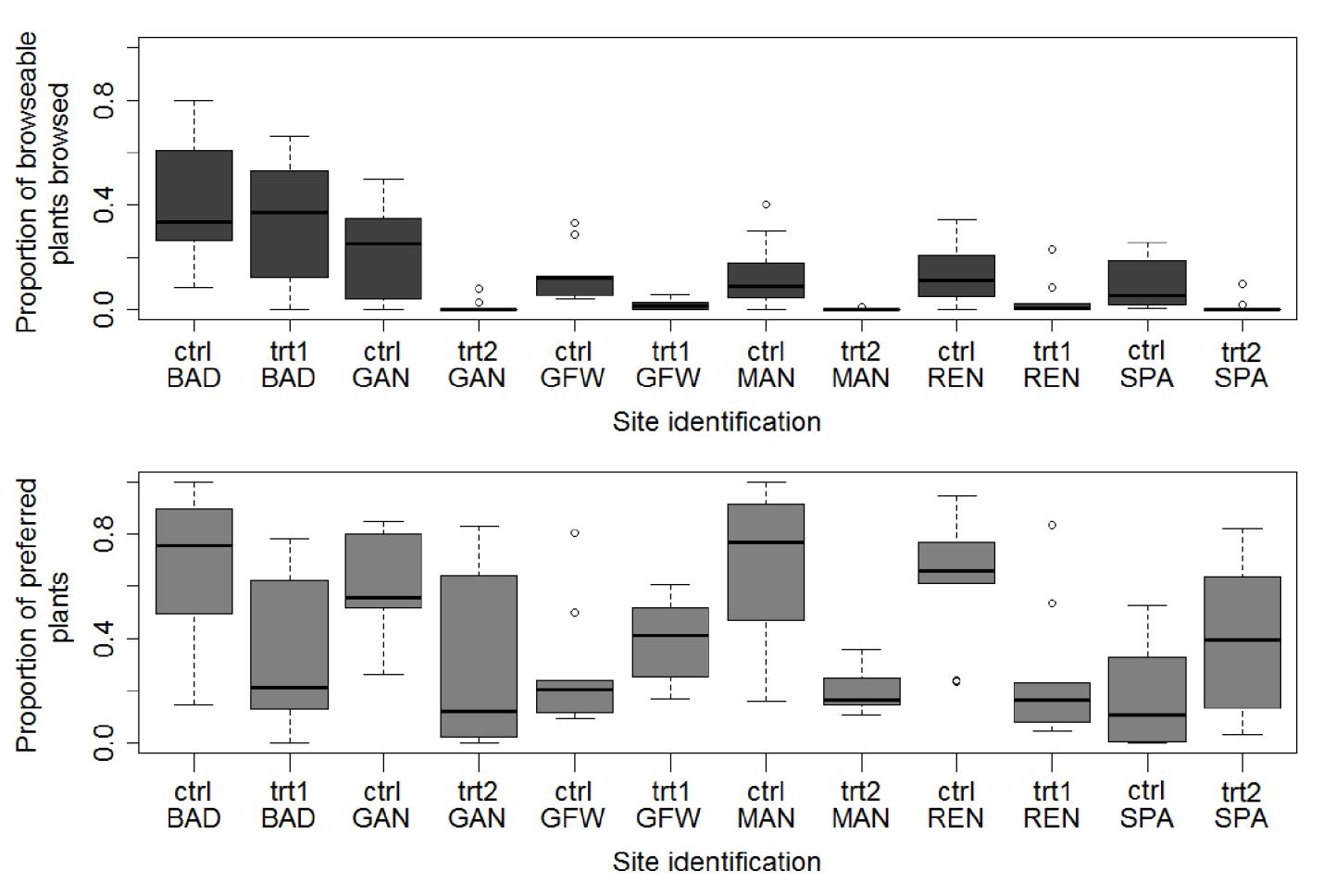


The proportion of moose browsed plants (dark gray) and the proportion of preferred plants (light gray) for each treatment (trt1 & trt2) and control (ctrl) site. Preferred plants are the 14 forage species that had significantly higher browse frequency as identified with our segmented regression analysis (see text for details). The locations; BAD: Badger, GFW: Grand Falls-Windsor, GAN: Gander Bay, MAN: La Manche Provincial Park, REN: Renews-Cappahayden, and SPA: Spaniards Bay.
