# Supplementary material for: Effect of Roadside Vegetation Cutting on Moose Browsing
Source: PLoS One. 2015 Aug 5;10(8):e0133155. doi: 10.1371/journal.pone.0133155 (PMC4526696; doi:10.1371/journal.pone.0133155)
Supplement: S2 Fig — Displaying the correlation between the proportion of preferred plants per plot and the 3 treatment types. There were higher quality plants present in the control areas than in the treatment areas (rho = −0.30, S = 272145.8, P = 0.002). Since we were testing for the effect that roadside vegetation cutting had on the proportion of moose browse in roadside areas (and through further AICc analysis), treatment type was used as the main explanatory variable rather than preferred plants. For the control and treatment groups: control sites: not cut since at least 2008, treatment 1 sites: cut from 2008–2010, and treatment 2 sites: cut from 2011–2013. For the locations; BAD: Badger, GFW: Grand Falls-Windsor, GAN: Gander Bay, MAN: La Manche Provincial Park, REN: Renews-Cappahayden, and SPA: Spaniards Bay. (DOCX) [file pone.0133155.s002.docx]

**S2 Figure. Correlation between the proportion of preferred plants per plot and the three treatment types.
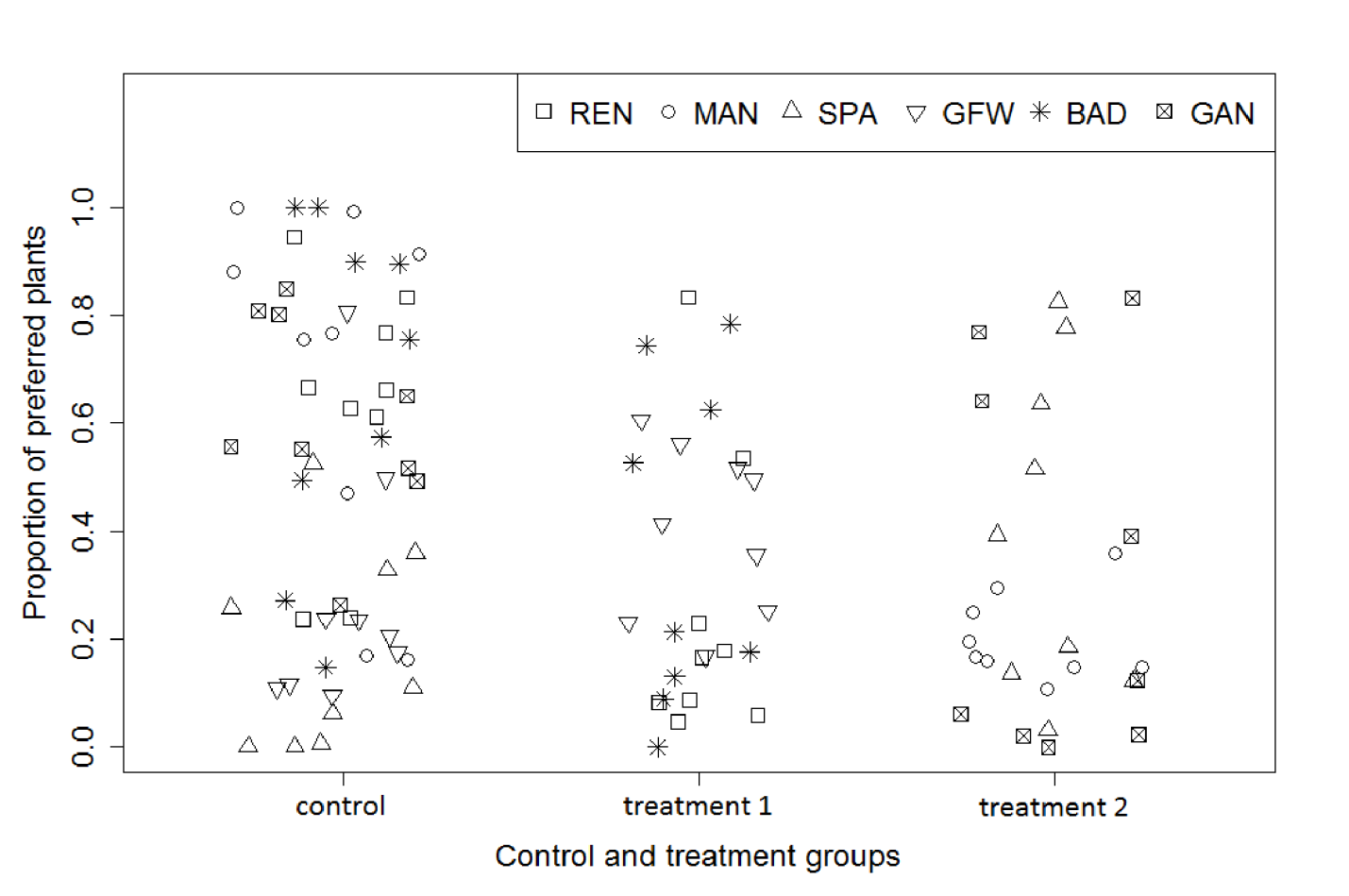
**

Displaying the correlation between the proportion of preferred or high quality plants per plot and the three treatment types. There were higher quality plants present in the control areas than in the treatment areas (*rho*=−0.30, S=272145.8, *P*=0.002). Since we were testing for the effect that roadside vegetation cutting had on the proportion of moose browse in roadside areas (and through further AIC_c_ analysis), treatment type was used as the main explanatory variable rather than preferred or high quality plants. For the control and treatment groups: control sites: not cut since at least 2008, treatment 1 sites: cut from 2008-2010, and treatment 2 sites: cut from 2011-2013. For the locations; BAD: Badger, GFW: Grand Falls-Windsor, GAN: Gander Bay, MAN: La Manche Provincial Park, REN: Renews-Cappahayden, and SPA: Spaniards Bay.
