# Supplementary material for: Effect of Roadside Vegetation Cutting on Moose Browsing
Source: PLoS One. 2015 Aug 5;10(8):e0133155. doi: 10.1371/journal.pone.0133155 (PMC4526696; doi:10.1371/journal.pone.0133155)
Supplement: S1 Table — Site description including; GPS locations, road speed limit, width, presence of water bodies, moose density and the gradient for both the road and tree sides of the site for the cut treatment (TRT) and uncut control (CRL) sites collected from June 17 –July 23 2014 in Newfoundland, Canada. For the locations; BAD: Badger, GFW: Grand Falls-Windsor, GAN: Gander Bay, MAN: La Manche Provincial Park, REN: Renews-Cappahayden, and SPA: Spaniards Bay. (DOCX) [file pone.0133155.s003.docx]

**S1 Table. General description of field study sites.**

Site description including; GPS locations, road speed limit, width, elevation, presence of water bodies, moose density and the gradient for both the road and tree sides of the site for the cut treatment (TRT) and uncut control (CRL) sites collected from June 17^th^ – July 23^rd^ 2014 in Newfoundland, Canada. For the locations; BAD: Badger, GFW: Grand Falls-Windsor, GAN: Gander Bay, MAN: La Manche Provincial Park, REN: Renews-Cappahayden, and SPA: Spaniards Bay.

|  | BAD TRT | BAD CRL | GFW TRT | GFW CRL | GAN TRT | GAN CRL | REN  TRT | REN  CRL | MAN TRT | MAN CRL | SPA  TRT | SPA  CRL |
| --- | --- | --- | --- | --- | --- | --- | --- | --- | --- | --- | --- | --- |
| **Latitude** | 48.945035 | 48.816628 | 49.008013 | 49.052005 | 49.348650 | 49.313023 | 46.851091 | 47.143087 | 47.203047 | 47.344274 | 47.605001 | 47.600894 |
| **Longitude** | −56.095413 | −56.582799 | −55.577648 | −55.592012 | −54.381017 | −54.433646 | −52.973544 | −52.901577 | −52.902267 | −52.915037 | −53.348765 | −53.342005 |
| **Year cut** | 2009 | pre-2008 | 2009 | pre-2008 | 2011 | pre-2008 | 2010 | pre-2008 | 2013 | pre-2008 | 2011 | pre-2008 |
| **Road speed** | 80 km/h | 80 km/h | 50 km/h | 60 km/h | 80 km/h | 80 km/h | 80 km/h | 80 km/h | 80 km/h | 80 km/h | 80 km/h | 50 km/h |
| **Site width** | 13.7 m | 13.7 m | 16.0 m | 16.0 m | 14.1 m | 14.1 m | 15.5 m | 15.5 m | 14.5 m | 14.5 m | 11.2 m | 11.2 m |
| **Elevation** | 120 m | 201 m | 95 m | 98 m | 40 m | 11 m | 43 m | 80 m | 66 m | 155 m | 105 m | 94 m |
| **Water body** | No | Yes | Yes | No | Yes | No | Yes | No | Yes | No | No | No |
| **Gradient road** | 0.50 | 0.60 | 0.54 | 0.55 | 0.70 | 0.55 | 0.50 | 0.58 | 0.59 | 0.21 | 0.57 | 0.44 |
| **Gradient tree** | 0.42 | 0.27 | No Slope | No Slope | No Slope | No Slope | No Slope | 0.67 | No Slope | 0.46 | 0.45 | 0.46 |
| **Traffic region** | Off Avalon | Off Avalon | Off Avalon | Off Avalon | Off Avalon | Off Avalon | On Avalon | On Avalon | On Avalon | On Avalon | On Avalon | On Avalon |
| **Moose density (moose/km^2^)** | 1.05 moose/km^2^ | 1.05 moose/km^2^ | 1.95 moose/km^2^ | 1.95 moose/km^2^ | 1.10 moose/km^2^ | 1.10 moose/km^2^ | 3.63 moose/km^2^ | 3.63 moose/km^2^ | 3.63 moose/km^2^ | 3.63 moose/km^2^ | 1.58 moose/km^2^ | 1.58 moose/km^2^ |
