# Supplementary material for: Effect of Roadside Vegetation Cutting on Moose Browsing
Source: PLoS One. 2015 Aug 5;10(8):e0133155. doi: 10.1371/journal.pone.0133155 (PMC4526696; doi:10.1371/journal.pone.0133155)
Supplement: S2 Table — Pictures of one of our control, treatment 1, and treatment 2 sampling sites, showing the width of the cut (in the treatment areas) and the height of the vegetation. A black arrow indicates the location of a person (height: 5’6” or 1.68 m) as a reference for the height of the vegetation. (DOCX) [file pone.0133155.s004.docx]

**S2 Table. Descriptions of the field sites for the three treatment types.**

Pictures of one of our control, treatment 1, and treatment 2 sampling sites, showing the width of the cut (in the treatment areas) and the height of the vegetation. A black arrow indicates the location of a person (height: 5’6” or 1.68 cm) as a reference for the height of the vegetation.

| 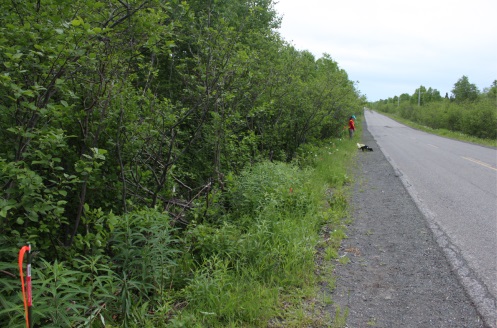 | Site 8: Control – uncut since at least 2008  Location: Grand Falls-Windsor  Cut width: matched to paired treatment (16 m) |
| --- | --- |
| 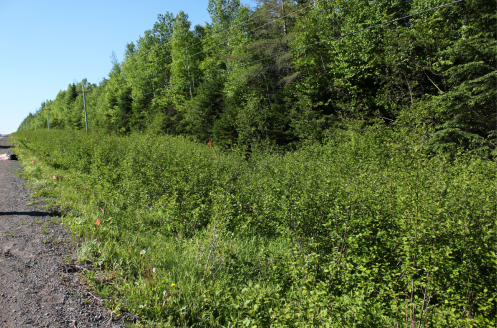 | Site 8: Treatment 1 – cut between 2008-2010  Location: Grand Falls-Windsor  Cut width: 16 m |
| 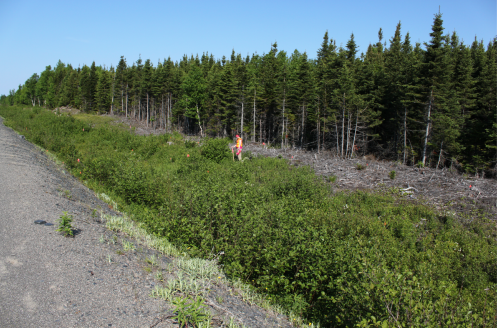 | Site 17: Treatment 2 – cut between 2011-2013  Location: Gander  Cut width: 14.1 m |
