# Supplementary material for: Effect of Roadside Vegetation Cutting on Moose Browsing
Source: PLoS One. 2015 Aug 5;10(8):e0133155. doi: 10.1371/journal.pone.0133155 (PMC4526696; doi:10.1371/journal.pone.0133155)
Supplement: S3 Table — Potential hypotheses for the effect that each explanatory variable would have individually on the proportion of moose browse in the roadside areas in Newfoundland, Canada. (DOCX) [file pone.0133155.s005.docx]

**S3 Table. Hypothesis of the effect of each explanatory variable on the proportion of moose browse along roadsides.**

Potential hypotheses for the effect that each explanatory variable would have individually on the proportion of moose browse in the roadside areas in Newfoundland, Canada.

| Explanatory variable | Explanatory Discrete or continuous variable | Effect on proportion of moose browse |
| --- | --- | --- |
| Treatment type | Discrete | The more recently the vegetation was cut, the greater the proportion of moose browse due to moose preferentially feeding on plant regrowth. |
| Water bodies | Discrete | Presence of water bodies would increase the proportion of moose browse due to moose being attracted to the water bodies to drink, feed on aquatic plants, and avoid insects [1]. |
| Traffic region | Discrete | The Avalon peninsula has higher traffic volumes and therefore more disturbance than central Newfoundland, resulting in avoidance of the roadside area by moose and a decrease in the proportion of browse. |
| Width of site | Continuous | The larger the width, the greater the proportion of moose browse due to the moose not having to venture as close to the road to feed. |
| Road speed limit | Continuous | The faster the speed limit, the greater the disturbance the traffic causes, resulting in a decrease in the proportion of moose browse. |
| Gradient up to the roadside | Continuous | The steeper the roadside gradient the greater the challenge for moose to maneuver out of the roadside area, resulting in the moose remaining in the roadside area and increasing the proportion of browse. |
| Gradient up to the tree-side | Continuous | The steeper the tree-side gradient, the greater the challenge for moose to maneuver into the roadside area, resulting in moose not entering into the roadside area, causing a decrease in the proportion of browse. |
| Moose density | Continuous | Higher moose density would increase the proportion of moose browse in roadside areas because a larger number of moose would live and forage in the area. |
| Plant preference index | Continuous | A higher proportion of preferred or high quality plants would increase in the proportion of moose browse in roadside areas due to the moose being attracted to the area to feed on preferred or high quality species. |

**References:**

1. Peek JM. Habitat relationships. In: Franzmann AW, Schwartz CC, editors. Ecology and management of the North American moose. 1st ed. Washington, D.C., USA: Smithsonian Institution Press; 1998. p. 351-75
