# Supplementary material for: Effect of Roadside Vegetation Cutting on Moose Browsing
Source: PLoS One. 2015 Aug 5;10(8):e0133155. doi: 10.1371/journal.pone.0133155 (PMC4526696; doi:10.1371/journal.pone.0133155)
Supplement: S4 Table — Pearson’s and Spearman’s correlation analyses were performed to determine which explanatory variables to include as fixed effects in the models. Pearson’s correlation was performed when both variables were continuous and Spearman’s correlation was performed when either one or both variables were discrete. The tolerance for Type 1 error was set at α = 0.05, therefore variables were considered correlated if the p-value was <0.05. (DOCX) [file pone.0133155.s006.docx]

**S4 Table. Pearson’s and Spearman’s correlation analyses of explanatory variables for moose browse along roadsides.**

Pearson’s and Spearman’s correlation analyses were performed to determine which explanatory variables to include as fixed effects in the models. Pearson’s correlation was performed when both variables were continuous and Spearman’s correlation was performed when either one or both variables were discrete. The tolerance for Type 1 error was set at α=0.05, therefore variables were considered correlated if the p-value was <0.05.

| Explanatory  variables | Spearman`s | | |
| --- | --- | --- | --- |
|  | rho | S | p-value |
| Treatment Year Group & Water Bodies | 0.48 | 109566.4 | 1.67e-07 |
| Treatment Year Group & Width of Cut | -0.12 | 234078.7 | 0.24 |
| Treatment Year Group & Road Speed | 0.25 | 158036.9 | 0.01 |
| Treatment Year Group & Road-side Gradient | 0.52 | 100039.6 | 6.14e-09 |
| Treatment Year Group & Tree-side Gradient | -0.41 | 296638.8 | 8.91e-06 |
| Treatment Year Group & Traffic Region | 0.08 | 193440.0 | 0.42 |
| Treatment Year Group & Moose Density | 0.00 | 209934.0 | 1.00 |
| Treatment Year Group & Site Quality | -0.30 | 272145.8 | 0.00 |
| Water Bodies & Width of Cut | 0.25 | 157988.8 | 0.01 |
| Water Bodies & Road Speed | 0.06 | 196400.4 | 0.51 |
| Water Bodies & Road-side Gradient | 0.17 | 173955.8 | 0.08 |
| Water Bodies & Tree-side Gradient | -0.55 | 325148.4 | 7.78e-10 |
| Water Bodies & Traffic Region | -0.17 | 245419.3 | 0.08 |
| Water Bodies & Moose Density | 0.05 | 199393.3 | 0.61 |
| Water Bodies & Site Quality | -0.13 | 236873.4 | 0.19 |
| Width of Cut & Traffic Region | -0.10 | 230421.5 | 0.32 |
| Road Speed & Traffic Region | 0.16 | 176573.3 | 0.10 |
| Road-side Gradient & Traffic Region | 0.05 | 199798.3 | 0.62 |
| Tree-side Gradient & Traffic Region | 0.52 | 101740.7 | 1.15e-08 |
| Traffic Region & Moose Density | 0.69 | 64428.06 | < 2.20e-16 |
| Traffic Region & Site Quality | -0.09 | 228453.0 | 0.36 |
| Explanatory  variables | Pearson`s | | |
|  | cor | t | p-value |
| Width of Cut & Road Speed | -0.01 | -0.13 | 0.90 |
| Width of Cut & Road-side Gradient | 0.12 | 1.26 | 0.21 |
| Width of Cut & Tree-side Gradient | -0.43 | -4.87 | 3.93e-06 |
| Width of Cut & Moose Density | 0.44 | 4.99 | 2.42e-06 |
| Width of Cut & Site Quality | 0.07 | 0.75 | 0.45 |
| Road Speed & Road-side Gradient | -0.05 | -0.46 | 0.64 |
| Road Speed & Tree-side Gradient | 0.08 | 0.87 | 0.38 |
| Road Speed & Moose Density | 0.18 | 1.89 | 0.06 |
| Road Speed & Site Quality | 0.24 | 2.53 | 0.01 |
| Road-side Gradient & Tree-side Gradient | -0.38 | -4.24 | 4.76e-05 |
| Road-side Gradient & Moose Density | -0.07 | -0.74 | 0.46 |
| Road-side Gradient & Site Quality | -0.29 | -3.11 | 0.00 |
| Tree-side Gradient & Moose Density | 0.21 | 2.21 | 0.03 |
| Tree-side Gradient & Site Quality | 0.26 | 2.72 | 0.01 |
| Moose Density & Site Quality | -0.01 | -0.07 | 0.94 |
