# Supplementary material for: Effect of Roadside Vegetation Cutting on Moose Browsing
Source: PLoS One. 2015 Aug 5;10(8):e0133155. doi: 10.1371/journal.pone.0133155 (PMC4526696; doi:10.1371/journal.pone.0133155)
Supplement: S5 Table — Two generalized linear mixed-effects models used to determine if treatment type or preferred plants were a better predictor of the proportion of browsed plants. The variables plot number and nested within site id were included as random effects in both models. (DOCX) [file pone.0133155.s007.docx]

**S5 Table. Glmer’s for comparison of treatment type and proportion of preferred plants.** Two generalized linear mixed-effects models used to determine if treatment type or preferred plants were a better predictor of the proportion of browsed plants. The variables plot number nested within site id were included as random effects in all of models.

| Model^a^ | Description | k^b^ | LL^b^ | Marginal *R*^2b^ | Conditional *R*^2b^ | ΔAIC_c_^b^ | ωAIC_c_^b^ |
| --- | --- | --- | --- | --- | --- | --- | --- |
| 1 | treatment type | 5 | -338.32 | 0.34 | 0.42 | 0.00 | 1.00 |
| 2 | proportion of  preferred plants 14spp | 4 | −361.30 | 0.11 | 0.24 | 43.96 | 0.00 |

^a^ Models are ranked with Akaike Information Criterion, corrected for small sample size (AIC_c_)

^b^ Key: k, number of parameters; LL, log-likelihood; Marginal *R*^2^, Nakagawa and Schielzeth’s Marginal *R*^2^ where the fixed factors alone explain the proportion of variance; Conditional *R*^2^, Nakagawa and Schielzeth’s Conditional *R*^2^ where both the fixed and random factors explain the proportion of variance; ΔAIC_c_, the difference in the AIC_c_; ωAIC_c_, model weights.
