# Supplementary material for: Effect of Roadside Vegetation Cutting on Moose Browsing
Source: PLoS One. 2015 Aug 5;10(8):e0133155. doi: 10.1371/journal.pone.0133155 (PMC4526696; doi:10.1371/journal.pone.0133155)
Supplement: S6 Table — Descriptive statistics of plant height in the sampling sites (CTRL: control—not cut since at least 2008, TRT 1: treatment 1 –cut between 2008–2010, and TRT 2: treatment 2 –cut between 2011–2013) in Newfoundland. The chart provides an overview of the structure of the plant community, including the proportion of plants in 3 height categories, and 1 combined category. The distinction at 30 cm was made because moose rarely browse below this height [49]. (DOCX) [file pone.0133155.s008.docx]

**S6 Table. Summary of plant height in the sampling sites.**

Descriptive statistics of plant height in the sampling sites (CTRL: control – not cut since at least 2008, TRT 1: treatment 1 – cut between 2008-2010, and TRT 2: treatment 2 – cut between 2011-2013) in Newfoundland. The chart provides an overview of the structure of the plant community, including the proportion of plants in 3 height categories, and 1 combined category. The distinction at 30 cm was made because moose rarely browse below this height [49].

|  | CTRL | TRT 1 | TRT 2 |
| --- | --- | --- | --- |
| Percent of plants  >200 cm | 8.10 | 2.59 | 0.27 |
| Percent of plants  30-200 cm | 59.12 | 50.18 | 48.84 |
| Percent of plants  <30 cm | 32.78 | 47.23 | 50.90 |
| Percent of plants  30-200 cm and >200 cm | 67.22 | 52.77 | 49.10 |

**References:**

49. Wam HK, Hjeljord O. Moose summer diet from feces and field surveys: a comparative study. Rangeland Ecol. Manage. 2010;63(3):387-95.
